# Impact of Urinary Incontinence and Toilet-Use Problems on People Living at Home With Dementia and Their Family Carers: A Descriptive Survey of Carer Perspectives in the UK

**DOI:** 10.1097/WON.0000000000001275

**Published:** 2026-05-05

**Authors:** Miriam Avery, Mandy Fader, Margaret Macaulay, Cathy Murphy

**Affiliations:** **Miriam Avery, PhD,** School of Health Sciences, University of Southampton, Southampton, UK.; **Mandy Fader, PhD,** School of Health Sciences, University of Southampton, Southampton, UK.; **Margaret Macaulay, MSc,** School of Health Sciences, University of Southampton, Southampton, UK.; **Cathy Murphy, PhD,** School of Health Sciences, University of Southampton, Southampton, UK.

**Keywords:** Behaviors that challenge, Carer, Dementia, Incontinence, Toilet use

## Abstract

**PURPOSE:**

This survey aimed to characterize carers’ perspectives on the impact of continence and toilet-use problems on people living at home with dementia and their family carers.

**DESIGN:**

Descriptive online survey.

**SUBJECTS AND SETTINGS:**

One hundred thirteen family carers completed the survey. Most were female (n = 89 [78%]) and adult children (n = 66 [58%]). Most were caring for someone with urinary and fecal incontinence

**METHODS:**

The survey was developed using existing data from qualitative interviews with family carers, people living with dementia and health care professionals and with input from stakeholders. Participants were family carers of people living at home with both dementia and continence care needs. Descriptive statistics and content analysis were used to summarize results.

**RESULTS:**

Around a half of carers thought that incontinence affected the health of the person they cared for and over one-third said it limited the person from taking part in social activities and seeing friends/family. The most common continence problems reported by carers were maintaining toilet-related hygiene, inappropriate removal of absorbent or other continence products, repetitive toilet behaviors/rituals, refusal to wear absorbent or other continence products, refusal to accept help with hygiene, and aggression related to continence care. Carers reported their biggest continence-related challenges to be increased workload, cost of pads, bad smell, lack of knowledge/support, arguments, and difficulties protecting dignity. Carers would like practical help with learning how to care for incontinence, adequate continence pad provision, and proactive sign-posting to continence services.

**CONCLUSIONS:**

Continence care can have a substantial and wide-ranging negative impact on people living at home with dementia and their carers. Strategies that can be tailored to individuals (both person with dementia and carer) to reduce the impact of these problems are urgently needed.

## INTRODUCTION

Most people living with dementia will develop problems with bladder or bowel continence or toilet use in the mid or late stages of illness, with 72% experiencing urinary incontinence in their final year.^1^ People living with dementia are at considerably higher risk (adjusted rate ratios: urinary incontinence 3.2 in men and 2.7 in women, fecal incontinence 6.0 for men and 4.5 for women) of developing incontinence than those without dementia.^2^ The causes of incontinence or toilet-use problems for people living with dementia are complex, commonly including a lack of insight into bodily sensations, problems with the process of reaching or using the toilet and functional limitations.^3,4^ Additionally, strategies that people without dementia use to manage these problems (eg, pre-emptive toilet use, pad use, regular pelvic floor exercises, or toilet-mapping) are challenged by the cognitive changes seen in persons living with dementia.

Incontinence can cause substantial harm to health and be hugely distressing, for both the person living with dementia and their carers.^2,3^ Dementia and incontinence may lead to social isolation^3^ or unwanted admission to a nursing home or care facility.^5^ Carers rate independent toilet use as the most important activity of daily living for the person living with dementia to preserve.^6^ Nevertheless, there is a long-reported lack of evidence or clinical guidance to support this group and their carers.^7,8^ Burholt and colleagues observed that limited research and the paucity of studies enrolling participants with dementia from continence care guidelines have marginalized this population, resulting in a belief that nothing can be done to manage continence problems in this population.^8^ There is an urgent need for co-produced interventions to meet the complex incontinence needs of people living with dementia and their carers.^8^ Given the lack of evidence or guidance, it is unsurprising that the support that people living with dementia and their carers have received to cope with continence management at home is unsatisfactory or, in some cases, non-existent. Dementia carers have long called for better guidance from health care professionals (HCPs) on how to cope.^2,3^ However, HCPs report that they lack knowledge about dementia-associated incontinence and feel frustrated by the lack of help they can provide.^3^ An added difficulty is that the stigma of incontinence causes delayed help-seeking.^9,10^

New interventions are needed to support carers of people living with dementia and continence problems. However, developing effective interventions for this group has proven challenging,^11^ and a systematic, theory-led approach to development is needed to improve the chances of achieving effective interventions to tackle the most pressing and bothersome problems. The first stage of intervention development is gaining a full and detailed understanding of the problem being tackled.^12^ Qualitative data describing continence and toilet-use problems for people living at home with dementia have been published.^3,4^ These data present a complex picture. To further characterize and start to quantify the problems faced, we needed data on a larger scale. The aim of this study was to characterize carers’ perceptions of the impact of continence and toilet-use problems and the provision of continence care on the lives of people living at home with dementia and their own lives.

## METHODS

A descriptive online survey was undertaken. A sequential exploratory approach^13^ was used with a 59-item online survey developed based on qualitative data from interviews with 45 people (26 unpaid carers, 2 people with dementia, and 9 continence and 8 dementia HCPs).^4^ Recruitment was via the Join Dementia Research website^14^ and took place over 3 months (December 2020 to February 2021). The study was advertised on the website once at the start of the recruitment period and additionally highlighted to carers who joined the website during the recruitment period. Adults aged 18 years and older who were (or had formerly been) a carer (unpaid family or friend) for someone with a diagnosis of dementia who had also experienced incontinence or toilet-use problems were invited to participate in the survey. Online participant information was provided to interested parties prior to study participation. The survey incorporated a consent statement at the start, requiring the participant to confirm that they had read and understood the participant information sheet and consented to take part in the study. The acknowledgment of the consent statement and the click finish at the end of the survey were taken as confirmation of consent.

This study was funded by an Alzheimer’s Society (UK) postdoctoral fellowship. Ethical approval was received from the University of Southampton (ERGO 55590).

### Instruments

Topics for survey questions were identified from published qualitative data,^3,4^ items were developed and reviewed by team members and stakeholders for face and content validity and tested for usability and acceptability by 2 patient and public involvement (PPI) contributors who were both former carers (see Appendix, Supplemental Digital Content 1, http://links.lww.com/JWOCN/A151). The item response options were dichotomous, multiple choice, scale-based, or open-ended free text covering the demographics of the carer and the person they cared for, carer experiences, and carer’s perceptions of the experiences of the person living with dementia. Open-ended options allowed respondents to provide explanations about how incontinence care had impacted the carer/person with dementia relationship, and to provide explanations when a response of “other” had been selected.

### Study Procedures

The survey was completed electronically using iSurvey software (iSurvey Group Billingstad, Norway). Data collection was undertaken during the COVID-19 pandemic and planned additional methods of data collection via community health care services were not feasible due to staff workload. The time taken to complete the survey was around 15 minutes based on testing by PPI contributors.

### Data Analysis

Simple descriptive statistics were employed to meet the aims of the study, including calculating ranges and proportions using Microsoft Excel. Qualitative analysis was undertaken on free-text responses using a summative content analysis approach without preconceived categories.^15^ This method is appropriate for summarizing simple free-text responses that comprised only a few words (eg, “more support from GP”). In its simplest form, this method can be used to identify, count, and group keywords. The Checklist for Reporting of Survey Studies (CROSS)^16^ was used to guide the reporting of this study.

## RESULTS

One hundred and thirteen respondents from across the UK completed the survey. Table 1 provides a summary of characteristics for both members of the care dyad. Not all respondents answered all questions and therefore responses do not always total 113, percentages are based on the proportion who answered the question. In around two-thirds (n = 73, 65%) of the care dyads, the person living with dementia was female. Ten (9%) were aged ≤70 years, 22 (19%) aged 71 to 80 years, and 81 (72%) were aged ≥81 years. A majority of respondents described themselves as white (n = 110, 97%). The carers were largely female (n = 89, 80%) and most (n = 76, 68%) were between 41 and 70 years of age.

**TABLE 1.** Characteristics of Carers and People With Dementia (as Reported by the Carers)

Characteristics of carers	N (%)
Sex (n = 111)	
Male	22 (20)
Female	89 (80)
Age in years (n = 112)	
40 or under	4 (4)
41-70	76 (68)
71-80	27 (24)
81 or over	5 (4)
Ethnicity (n = 113)	
White British	110 (97)
Asian/Asian British	2 (2)
Black/African/Caribbean/Black British	0
Other ethnicity	1 (1)
Past or current carer at time of survey (n = 113)	
Past	35 (31)
Current	78 (69)
Relationship to person with dementia (n = 110)	
Spouse/partner	35 (32)
Adult child	66 (60)
Sibling	1(1)
Friend	3 (3)
Daughter-in-law	2 (2)
Grandchild	2 (2)
Ex-spouse	1 (1)
Domestic arrangements (n = 108)	
Lives (or lived) with person with dementia	63 (58)
Lives (or lived) separately from person with dementia	45 (42)
**Characteristics of people with dementia (as reported by carers)**	**N (%)**
Sex (n = 113)	
Male	40 (35)
Female	73 (65)
Age (n = 113)	
70 or under	10 (9)
71-80	22 (19)
81 or over	81 (71)
Ethnicity (n = 113)	
White British	109 (96)
Asian/Asian British	2 (2)
Black/African/Caribbean/Black British	1 (1)
Other ethnicity	1 (1)
Type of dementia (n = 105)	
Alzheimer’s disease	48 (46)
Vascular	15 (14)
Mixed	31 (29)
Lewy body	4 (4)
Frontotemporal	1 (1)
Other	6 (5)
Stage of dementia (n = 107)	
Early	8 (7)
Mid	41 (38)
Late	58 (54)
Paid carers in home (n = 113)	
Yes	66 (58)
No	47 (42)
Type of incontinence (n = 113)	
Urinary only	30 (27)
Fecal only	5 (4)
Both urinary and fecal	78 (69)

Sixty-three respondents (58%) stated that they lived with the person with dementia and 45 (42%) did not. Of those who did not, 10 (22%) saw the person they cared for more than once per day, 8 (18%) once per day, 13 (29%) most days, 12 (27%) 1 to 3 times per week, and 2 (4%) less than once per week. Eighty-five (75%) provided at least weekly help with hygiene care. Sixty-six (58%) of the people living with dementia had paid carers at home. Of those who had paid carers, 3 (5%) had live-in carers, 47 (71%) had visits once or more per day (with 19 [29%] 3 or more times per day), and the remainder had visits 1 to 6 times per week. Out of the 57 respondents who answered the question “Have incontinence problems changed over the time the person you care for has suffered from dementia?,” 55 (96%) said that it had become more of a problem. Nine (8%) of the care recipients people had problems with urinary incontinence (that were known to the respondent) before developing dementia, and 1 (1%) had both urinary and fecal incontinence prior to a dementia diagnosis. Of those who responded, 80% (59/73) reported that the person they cared for experienced urinary incontinence every or most days, with 43% (32/73) experiencing fecal incontinence every or most days (Table 2). When asked why the person that they cared for experienced continence problems (giving as many reasons as relevant), 68 (62%) thought that the person sometimes knew that they needed to empty their bladder or bowels, but could not reach the toilet in time, 63 (57%) thought that they did not always know that they needed to use the toilet, 38 (35%) said that the person could not remember where the toilet was, 42 (38%) stated that the person did not remember the toilet-use processes such as removing clothing before sitting down, and 25 (23%) stated that the person had a physical problem such as prostate or prolapse issue. Several carers reported that hospital admission led to the start of continence problems.

**TABLE 2.** How Often Did the Person Experience Incontinence, N (%)

Type of Incontinence	Every Day	Most Days	1-3 Times/Week	Less Than Once/Week	Less Than Once/Month
Urinary	47 (64)	12 (16)	6 (8)	5 (7)	3 (4)
Fecal	17 (23)	15 (20)	19 (26)	12 (16)	10 (14)

Carers were asked how they thought that incontinence and its care affected the person they were caring for. Based on carers’ perceptions, the activities of the care recipient that were most impacted by continence problems were going away for the night, going on holiday, social activities (eg, groups or clubs), or seeing friends/family (with between a third and 40% impacted to a “great extent” or “somewhat” for each activity; Table 3). Other activities that carers mentioned in the free text included using the transport to the day center, going for a day out (eg, to the beach), swimming, cycling, going for a drive, or being able to stay at home by themselves. Of those who responded, around half thought that incontinence sometimes made the person that they cared for embarrassed or upset, with just under a fifth reporting that their care recipient worried about smell (Table 4). Half (55/109) of carers thought that incontinence affected the health of the person they cared for, around a fifth (23/109) did not know, and less than a third thought that it had not (31/109). Common health consequences included sore skin (n = 31, 28%), reduced sleep (n = 21, 19%), reduced social life (n = 14, 13%), increased anxiety (n = 39, 36%), UTI (n = 9, 8%), and limiting fluids (n = 23, 21%). The majority (n = 77, 71%) of carers reported that incontinence had a negative impact on the person’s home. The main damage described was to carpets and furniture, including beds and chairs.

**TABLE 3.** Carers’ Perceptions of How Much Continence Problems Impact the Activities of the Person They Care for, N (%)

	To a Great Extent	Somewhat	Very Little	Not at All	Not Applicable
Seeing friends or family (n = 109)	10 (9)	24 (22)	18 (16)	42 (38)	15 (14)
Going shopping (n = 109)	19 (17)	25 (23)	10 (9)	16 (15)	39 (36)
Working (n = 108)	6 (6)	4 (4)	1 (1)	7 (6)	90 (83)
Social activities (eg, attending groups or clubs; n = 108)	13 (12)	27 (25)	17 (16)	17 (16)	34 (31)
Having visitors at home (n = 108)	8 (7)	11 (10)	19 (18)	56 (52)	14 (13)
Going away for the night (n = 108)	28 (26)	9 (8)	3 (3)	11 (10)	57 (53)
Going on holiday (n = 107)	31 (29)	9 (8)	2 (2)	11 (10)	54 (50)

**TABLE 4.** Carers’ Perceptions of the Person With Dementia Experiencing Embarrassment or Worry Related to Incontinence, N (%)

Does the Person Ever	Sometimes	Seldom	Never	Don’t Know
Feel embarrassed or upset (n = 68)	35 (51)	15 (22)	13 (19)	5 (7)
Worry about smell (n = 94)	17 (18)	20 (21)	41 (44)	16 (17)

When they became carers, only around half (n = 58, 53%) were aware that the person might develop continence problems; the rest were either unaware (n = 44, 40%) or unsure (n = 8, 7%). Between a half and a third of those who responded reported that the continence problems of the person they cared for impacted them as a carer; for example, going on holiday (55/110, 50%), going away for the night (46/109, 42%), or seeing friends/family (36/110, 33%) to a “great extent” or “somewhat.” Around a quarter said that the problems impacted working (24/110, 22%), going shopping (27/110, 24%), and social activities (31/109, 28%; Table 5). In the free text, other activities that carers said were impacted included sleep, leaving the house and hobbies.

**TABLE 5.** Carers’ Perceptions of How Much the Continence Problems of the Person They Care for Impact Their Own Activities

	To a Great Extent	Somewhat	Very Little	Not at All	Not Applicable
Seeing friends or family (n = 110)	12 (11)	24 (22)	13 (12)	53 (48)	8 (7)
Going shopping (n = 110)	9 (8)	18 (16)	17 (15)	56 (51)	10 (9)
Working (n = 110)	12 (11)	12 (11)	9 (8)	38 (34)	39 (35)
Social activities (eg, attending groups or clubs; n = 109)	11 (10)	20 (18)	17 (16)	47 (43)	14 (13)
Having visitors at home (n = 109)	8 (7)	10 (9)	17 (16)	62 (57)	12 (11)
Going away for the night (n = 109)	30 (27)	16 (15)	6 (5)	35 (32)	22 (20)
Going on holiday (n = 110)	35 (32)	20 (18)	4 (4)	31 (28)	20 (18)

When asked to state what their main continence concerns about going out with the person with dementia were, the following common topics were identified: (1) lack of access to toilets; (2) fear of having an “accident”—mess and embarrassment; (3) careful planning needed, eg, change of clothes, where to park; (4) timing of trips, clock watching; (5) having to go into the toilets of the opposite sex if accessible toilets were not available; (6) stress of “second guessing” toilet needs—constant vigilance required; and (7) incontinence prevented trips from taking place.

When asked about a range of continence-related problems, the one that most frequently affected the carer with over two-thirds (75/109, 68%) being affected was maintaining toilet-related hygiene (eg, wiping). Around 40% were affected by inappropriate removal of absorbent or other continence products (51/109, 46%), repetitive toilet behaviors/rituals (49/107, 45%), refusal to wear absorbent or other continence products (40/110, 36%), refusal to accept help with toilet-related hygiene (47/109, 43%), and aggression related to continence or toileting care (42/109, 39%; Table 6). In free text, other common problems highlighted by carers were (1) denial or lack of insight related to incontinence, (2) no training or help, (3) increased workload (eg, laundry), (4) waking at night to urinate, and (5) the person altering their fluid intake or diet to try to avoid continence problems.

**TABLE 6.** Carers’ Perceptions of How Much Different Continence-Related Problems Have Affected Them, N (%)

	To a Great Extent	Somewhat	Very Little	Not at All
Refusal to wear pads or other continence products (n = 110)	13 (12)	27 (24)	16 (15)	54 (49)
Inappropriate removal of pads or other continence products (n = 109)	23 (21)	28 (25)	21 (19)	37 (34)
Inappropriate disposal of pads (n = 108)	20 (18)	25 (23)	14 (13)	49 (45)
Repetitive toilet behavior or rituals (n = 107)	28 (26)	21 (19)	13 (12)	45 (42)
Urinating in the “wrong” place (n = 109)	18 (16)	19 (17)	22 (20)	50 (46)
Defecating in the “wrong” place (n = 109)	12 (11)	14 (13)	21 (19)	62 (57)
Problems with maintaining toilet-related hygiene (eg, wiping; n = 109)	32 (29)	43 (39)	16 (15)	18 (16)
Refusal to accept help with toilet-related hygiene (n = 109)	15 (14)	32 (29)	25 (23)	37 (34)
Aggression related to receiving incontinence or toileting care (n = 109)	17 (16)	25 (23)	24 (22)	43 (40)

Additionally, 60% (65/110) of respondents said that the incontinence problems had made them feel upset or embarrassed “often” or “sometimes” and 64% (70/110) said that they “often” or “sometimes” worried about smell (Table 7). Forty-seven carers thought that incontinence affected their relationship with the person they cared for; however, 63 (57%) did not. Of those who did, the reasons given for this change included: (1) frustration caused by constant assistance needed with toilet use (including related activities such as wiping or hand washing); (2) distress experienced by one or both due to continence related embarrassment/shame; (3) feeling more like a carer than spouse/child, (4) continence problems or associated care strategies were the cause of arguments; (5) intimate nature of continence care was difficult for one or both, including resentment at having to provide intimate care; (6) exhaustion from providing continence care; (7) sadness at continence problems being a visible/tangible sign of decline; (8) feelings of being unable to cope or lack of self-efficacy; (9) lack of catheter care guidance; (10) how to manage own (carer) emotions; and (11) dealing with aggression.

**TABLE 7.** Carer Reports of Embarrassment or Worry Related to the Care Recipient’s Incontinence, N (%)

Do You Ever	Often	Sometimes	Never
Feel embarrassed or upset about their incontinence (n = 110)	27 (25)	38 (35)	24 (22)
Worry about smell related to their incontinence (n = 94)	33 (30)	37(34)	18 (16)

Nearly 4 out of 5 carers stated that incontinence had an impact on their stress levels (87/110, 79%), with around two-thirds reporting that it affected their emotional state (79/110, 71%) or saying that their time for themselves was affected (67/108, 61%). Almost half said that their physical state was affected (51/110, 47%; Table 8). Around one-third (35/109, 32%) of carers found it difficult to talk to the person with dementia about incontinence, and the same number struggled to speak to friends or family (35/109, 32%). In contrast, only 6 (6/109, 5%) found it difficult to speak to HCPs about the topic.

**TABLE 8.** Impact of Incontinence on Carers, N (%)

Has the Incontinence Had (or Did the Incontinence Have) Any Impact on the Following	To a Great Extent	Somewhat	Very Little	Not at All
Your finances (n = 110)	9 (8)	22 (20)	34 (31)	45 (41)
Your emotional state (n = 110)	40 (36)	39 (35)	22 (20)	9 (8)
Your stress levels (n = 110)	47 (43)	40 (36)	20 (18)	3 (3)
Your physical state (n = 110)	14 (13)	38 (34)	32 (28)	26 (24)
Time for yourself (n = 108)	29 (26)	38 (25)	24 (22)	17 (15)

When asked to describe, in their own words, the continence problem that caused the most concern, carers gave responses that have been summarized in the following categories: (1) lack of support (including lack of advice about or assistance obtaining absorbent products from health provider); (2) arguments with the person and changes in relationship; (3) negative emotions about continence, including managing stress and trying to remain calm; (4) increased workload related to laundry and cleaning; (5) not knowing what to do; (6) dementia-related behaviors, such as removal of pads; (7) struggling to protect the person’s dignity; (8) maintaining hygiene; (9) damage to home; (10) dealing with fecal leaks; (11) cost of absorbent products, new towels, replacing furniture, etc; (12) stopping socializing and going out; and (13) smell.

Approximately half of carers (56/109, 51%) received advice on managing incontinence (10 [9%] from a GP, 2 [2%] from a nurse working in general practice, 15 [14%] from a community nurse, 43 [39%] from a continence nurse, 10 [9%] from a dementia nurse, 1 [1%] from a pharmacist, 16 [15%] from paid carers, 3 [3%] from a friend, 1 [1%] from the Alzheimer’s Society helpline, and 1 [1%] from a pad manufacturer helpline). Twenty-eight (27%) also got information from the internet, 8 (7%) from leaflets, and 2 (2%) from books. Some had previous experience as a carer or nurse to draw upon. A common theme was that “any” information on how to cope would have been welcome and that HCPs did not proactively provide support. Additionally, incontinence affected some people’s access to services with 15 (14%) carers saying that access to day centers, other health care services (eg, journey to clinics was too long), and voluntary support services were impacted.

Support that carers would like to see included help with the steep learning curve, instructions and guidance, enough pads, practical training, and how to access continence services. Free-text responses indicated that many carers would like an HCP to provide information on (1) strategies for coping with continence problems, for example, managing hygiene and advice on products to use (including bed protection) and (2) opportunities to talk through issues, for example, advice on problems to look out for (eg, skin irritation or urinary tract infections) or receiving signposting to any available help. Forty-one (38%) would have liked help from a care (health or social) professional in person, 22 (20%) via a telephone helpline, 21 (19%) would like online information and 17 (16%) would have liked a booklet.

## DISCUSSION

In this article, we present data from a survey reporting on the experiences of carers supporting someone with both dementia and continence problems. We were unable to identify any published studies reporting the results of any similar survey conducted on the same scale. We therefore assert that survey findings provide much needed characterization of carers’ perceptions of the impact that continence problems and continence care on both them and the person for whom they are caring. Findings indicate that continence management causes a substantial negative impact on carers’ stress levels and emotional state, giving useful insight into the wide-ranging and often highly distressing needs and experiences of carers.

The survey sample is representative of the wider population in some respects. Both the higher number of female to male carers^17^ and the profile of types of dementia^18^ largely reflect the broader population. However, the person living with dementia and spouse dyadic relationships were underrepresented in this study, with the person and adult child relationship overrepresented. This is likely due to the recruitment method. Like many dementia studies, the number of participants from ethnic minorities is also underrepresented.^19^

This work emphasizes that continence problems for people living with dementia and their carers are not homogeneous; they are wide-ranging and different for every dyad. In common with qualitative studies on the topic, both the emotional impact of continence problems and the many practical issues that need to be addressed were repeatedly highlighted.^3,4^ Importantly, this study builds on qualitative work^3^ to shed further light on the relative frequency and level of impact of the different problems. The most frequently experienced problem was managing hygiene needs, followed by refusal to wear absorbent products, removal of absorbent products, repetitive toilet behaviors, refusal to accept help, and aggression related to continence care. For most carers, these problems caused a negative impact on their physical and mental health. It is worth highlighting that, despite the devastating impact of continence problems, most respondents reported that coping with these problems did not affect the dyadic relationship. For those whose relationship was affected, this was largely due to negative emotions including sadness, frustration and embarrassment, or feelings of being unable to cope with continence care demands.

These new insights indicate that any interventions should be tailorable to individual needs and circumstances. This reflects findings from studies in other areas of dementia care that have stated that to improve the chances of being effective, interventions should consider both the complexities of living with dementia^11^ and focus on the matching carer needs.^20^ Our findings indicate that key areas for intervention development are likely to include those aimed at increasing carer practical knowledge; improving ability to manage negative emotions such as shame, anger, or embarrassment; improving decision-making around, and access to, assistive products (including those to help protect the home), and managing behaviors that challenging behaviors. This necessity for interventions to be tailorable to individuals’ circumstances reiterates the previously observed requirement for a holistic needs-assessment process specifically designed to meet the continence needs of people living with dementia at home and their carers.^21^

This work also adds to the body of work highlighting the stigmatizing nature of continence problems, particularly with this population, and the challenges that this can pose for improving health literacy and engagement with health care practitioners.^22,23^ The survey findings show that the majority of respondents wanted “in-person” support from an HCP, including provision of information and opportunities to discuss issues. Informal carers have previously identified a preference for receiving information from their health care providers, considering them a trusted source of information and more “authoritative.”^22^ They also identified the need for information about possible future problems and ways they might be prevented.

### Limitations

This study has several limitations. Only 4 respondents were from ethnic minorities, with 109 white British (echoing what has been found in previous studies and representing an issue that needs to be addressed in research in the UK), and there were more adult child than spousal carer respondents. Second, not all respondents answered all questions. In addition, data were collected during the COVID-19 pandemic, and this might have influenced findings. Finally, there is likely to have been some conflation between the impact of continence problems and other dementia-related problems.

Nonetheless, this study benefits from being developed from in-depth interviews with carers of people living with dementia, and it offers insight supporting the characterization of continence care problems faced by this population. We assert that study findings support the development of interventions aimed at reducing the continence-associated workload.

### Implications for Research and Clinical Practice

Although undertaken in the UK, it is likely that these findings are relevant elsewhere, particularly in other developed countries with similarly high levels of dementia, as the problems faced are not specific to one health care system. Further research on the views of people living with dementia is now needed to complement these findings. There are many variables that warrant further examination, including the impact of the dyad dynamic (eg, adult, spouse or other carer, and male or female care recipient), type and stage of dementia, other comorbidities, and type and level of incontinence. These variables can affect, for example, the degree of burden experienced by the carer or their help-seeking behaviors.^22,24,25^ A larger scale survey to enable comparisons between groups and examine the impact of different types of problems for those who experience them would be beneficial in supporting focused intervention development and delivery. Pending the findings of future research, nurses (particularly specialist continence or dementia nurses) supporting people living with dementia and their carers should undertake a holistic continence assessment to fully understand the range of problems they are facing. Given the lack of support and information reported by carers, as a minimum, sign-posting to practical guidance (eg, www.demcon.org.uk—an evidence-based website on continence and dementia) should be provided.

## CONCLUSIONS

Managing continence problems can have a substantial negative impact on carers and the person living with dementia they are caring for. Nurses should recognize that the causes, impact, and degree of impact vary widely. Theoretically-driven, person-centered strategies that can be tailored to individual circumstances to reduce the impact of these problems on both the person living with dementia and their carers are urgently needed. Interventions that focus on negative emotions and stress related to continence care and challenging behaviors associated with hygiene, products, and maintaining toilet use have been identified as priorities.
